# JRTE-GCN: A Graph Convolutional Network with Joint Relation Topology Evolution Modeling for Skeleton-Based Action Recognition

**DOI:** 10.3390/jimaging12090420

**Published:** 2026-09-06

**Authors:** Qun You, Shaoqi Zhang, Xiaoyu Zou, Jinhao Chen, Yizhuo Zhang

**Affiliations:** 1 School of Computer Science and Artificial Intelligence, Aliyun School of Big Data, School of Software, Changzhou University, Changzhou 213164, China; 2 Academy for Educational Development and Innovation, The Education University of Hong Kong, 10 Lo Ping Road, Tai Po, New Territories, Hong Kong, China; 3 Hi-Think Technology Corp., Changzhou 213000, China

**Keywords:** skeleton-based action recognition, graph convolutional network, joint relation topology evolution, dynamic topology, persistent homology, human motion understanding, computer vision

## Abstract

Graph convolutional networks (GCNs) have become a mainstream approach for skeleton-based action recognition by modeling spatial dependencies among human joints. However, existing methods mostly rely on learnable adjacency matrices or average relations over the entire action sequence to construct graph structures. Such designs mainly represent the overall static structure of the skeleton and may be insufficient for characterizing dynamic changes in joint relations during action execution. To address this problem, this paper proposes a graph convolutional network with joint relation topology evolution modeling, termed JRTE-GCN. Specifically, the proposed method first constructs a topology evolution matrix from frame-level joint distance sequences by calculating the temporal standard deviation and the difference between the initial and final stages of an action. This matrix reflects both the fluctuation intensity and stage-wise variation of joint relations. Subsequently, persistent homology is used to extract structural features from the matrix, thereby capturing the dynamic evolution of joint relations. Finally, the extracted evolutionary features are transformed into layer-wise biases and fused with the average topological bias through a learnable residual mechanism. The fused biases are incorporated into the graph convolutional feature extraction process, enabling joint modeling of the overall skeleton structure and dynamic relational changes. Experimental results on NTU RGB+D 60, NTU RGB+D 120, and Northwestern-UCLA show that JRTE-GCN achieves competitive recognition accuracy under multiple evaluation protocols, demonstrating the effectiveness of the proposed method.

## 1. Introduction

The research focus of skeleton-based action recognition has gradually shifted from pure temporal feature extraction to the direct modeling of structural relations among human joints. Early methods employed recurrent neural networks (RNNs) to model the temporal dependencies of joint sequences, but they had difficulty effectively capturing the complex spatial structural relations among human joints. More importantly, during real-world action execution, the relations among joints are not fixed, but exhibit temporal fluctuations and stage-wise differences as the action proceeds. However, existing methods mostly rely on fixed physical skeletal connections or average relations over the entire action sequence, which may ignore such dynamic changes during action execution. Therefore, accurately capturing the dynamic changes in joint relations and extracting discriminative features for distinguishing different actions remain major challenges in this field.

Graph convolutional networks (GCNs) can aggregate node features on non-Euclidean skeletal graphs and have become a major approach for modeling spatial joint structures. However, existing GCN-based methods usually construct graph structures based on fixed physical skeletons, learnable adjacency matrices, or average relations over the entire sequence. These methods essentially reflect the overall structure of the whole action sequence. In practical action execution, however, the relations among joints change continuously. Static or averaged graph representations are therefore not sufficiently flexible and may be insufficient for accurately characterizing the temporal evolution of joint relations.

To overcome the above limitations, this paper proposes JRTE-GCN, a graph convolutional network based on joint relation topology evolution modeling. While preserving the average joint relations over the entire action sequence, the proposed method introduces the dynamic changes of joint relations during action execution. Specifically, we first construct sequences of pairwise joint distances frame by frame, and then calculate their temporal standard deviations and the differences between the initial and final stages of the action, thereby constructing a topology evolution matrix that reflects action-related relational changes. Subsequently, persistent homology is used to extract structural features from this matrix during a multi-threshold filtration process. Finally, the extracted evolutionary features are mapped into layer-wise biases and fused with the average topological bias in the form of learnable residuals, which are then incorporated into each graph convolutional backbone layer. This design enables the network to utilize both the overall average structure of the skeleton and the dynamic change information during action execution when updating features layer by layer.

The main contributions of this paper are summarized as follows:

1. We propose JRTE-GCN, a graph convolutional network based on joint relation topology evolution modeling. By introducing relation evolution information beyond the average joint relation topology, the proposed method alleviates the insufficient representation ability of average topology descriptions for dynamic structural changes.
2. We construct a joint relation topology evolution matrix. This matrix is computed from frame-level pairwise joint distance sequences and contains both full-sequence relational fluctuations and differences between the initial and final stages of an action, thereby representing the dynamic changes in joint relations.
3. We design a topology evolution bias fusion mechanism. This mechanism uses persistent homology to encode the topology evolution matrix and maps the obtained features into layer-wise topological biases, which are fused with the average topological bias in the form of learnable residuals and then incorporated into each graph convolutional backbone layer.
4. We validate the effectiveness of JRTE-GCN on the NTU RGB+D 60, NTU RGB+D 120, and Northwestern-UCLA datasets. Experimental results show that the proposed method achieves favorable recognition performance under multiple evaluation protocols, verifying the effectiveness of joint relation topology evolution modeling.

## 2. Related Work

### 2.1. Skeleton-Based Action Recognition via Sequence and Convolutional Modeling

Early skeleton-based action recognition methods usually represented human skeletons as joint-coordinate sequences and employed recurrent neural networks to model temporal dependencies. Representative studies modeled hierarchical body-part motion, spatial-temporal joint dependencies, attention-based key joints and frames, and view-adaptive representations [1,2,3,4]. Although these methods can capture temporal variations in skeleton sequences, their recurrent computation limits parallel efficiency, and the structural relations among joints are usually modeled only implicitly.

To improve feature extraction efficiency, some studies reorganized skeleton sequences into images, trajectory maps, feature maps, or heatmap volumes, enabling convolutional networks, attention models, and 3D CNNs to learn skeleton representations [5,6,7,8,9,10]. However, mapping irregular skeletal structures into regular grid-like representations may weaken the original topological relations and long-range joint dependencies. Therefore, subsequent studies have increasingly turned to graph-based modeling, in which human joints are treated as nodes and their relations are modeled as edges.

### 2.2. Graph-Based Joint Relation Modeling

Graph convolutional networks (GCNs) have become a mainstream framework for skeleton-based action recognition by modeling joints as nodes and skeletal connections or learned relations as edges. ST-GCN first formulated skeleton sequences as spatial-temporal graphs based on physical skeletal connections and temporal links across consecutive frames [11]. However, predefined skeletal graphs mainly propagate information along fixed bones, which limits their ability to capture long-range and action-specific joint dependencies.

To improve graph flexibility, subsequent studies introduced adaptive, dynamic, directed, multi-scale, and efficient graph modeling strategies. These methods extend fixed skeletal topology through learnable or sample-dependent adjacency structures, action-related relations, dynamic topology learning, direction-aware modeling, multi-scale spatial-temporal representation, and shift-based feature interaction [12,13,14,15,16,17]. More recent studies have further enhanced graph-based skeleton representation from the perspectives of channel-wise topology learning, discriminative feature learning, efficient architecture design, and hierarchical human structure modeling [18,19,20,21]. Overall, existing GCN-based methods have evolved from fixed skeletal topology to adaptive and multi-scale relation modeling. Nevertheless, most of them adjust graph structures implicitly during feature propagation and do not explicitly model the temporal evolution of skeletal topology, making it difficult to distinguish graph-structure variations across different action stages.

### 2.3. Topological Modeling Methods in Skeleton-Based Action Recognition

In addition to graph convolution-based joint relation modeling, some studies have explored topological information for skeleton action representation. Persistent homology has been used to characterize connectivity relations and their stability, providing global structural information that complements joint coordinates and motion trajectories [22,23]. Other methods have incorporated skeletal topology into convolutional design, modeled relations among multiple joint variables, or introduced graph distance and persistent homology into GCN-based skeleton representation [24,25,26]. These studies show that topological information can complement existing skeleton representations from both global structure and joint organization perspectives.

However, existing topological methods mainly focus on the overall skeleton structure or aggregated topological features over the entire sequence. Such representations can describe average joint relations, but they may overlook temporal changes in joint relations during action execution. For actions involving stage transitions, limb coordination, or human–object interaction changes, average relations may be insufficient to distinguish structural differences across action stages. To address this limitation, JRTE-GCN explicitly models joint relation topology evolution from frame-level joint relations, extracts topological features using persistent homology, and fuses the resulting topology evolution bias with the average topological bias to enhance graph convolutional feature learning.

## 3. Materials and Methods

### 3.1. Overview

**Figure 1:**
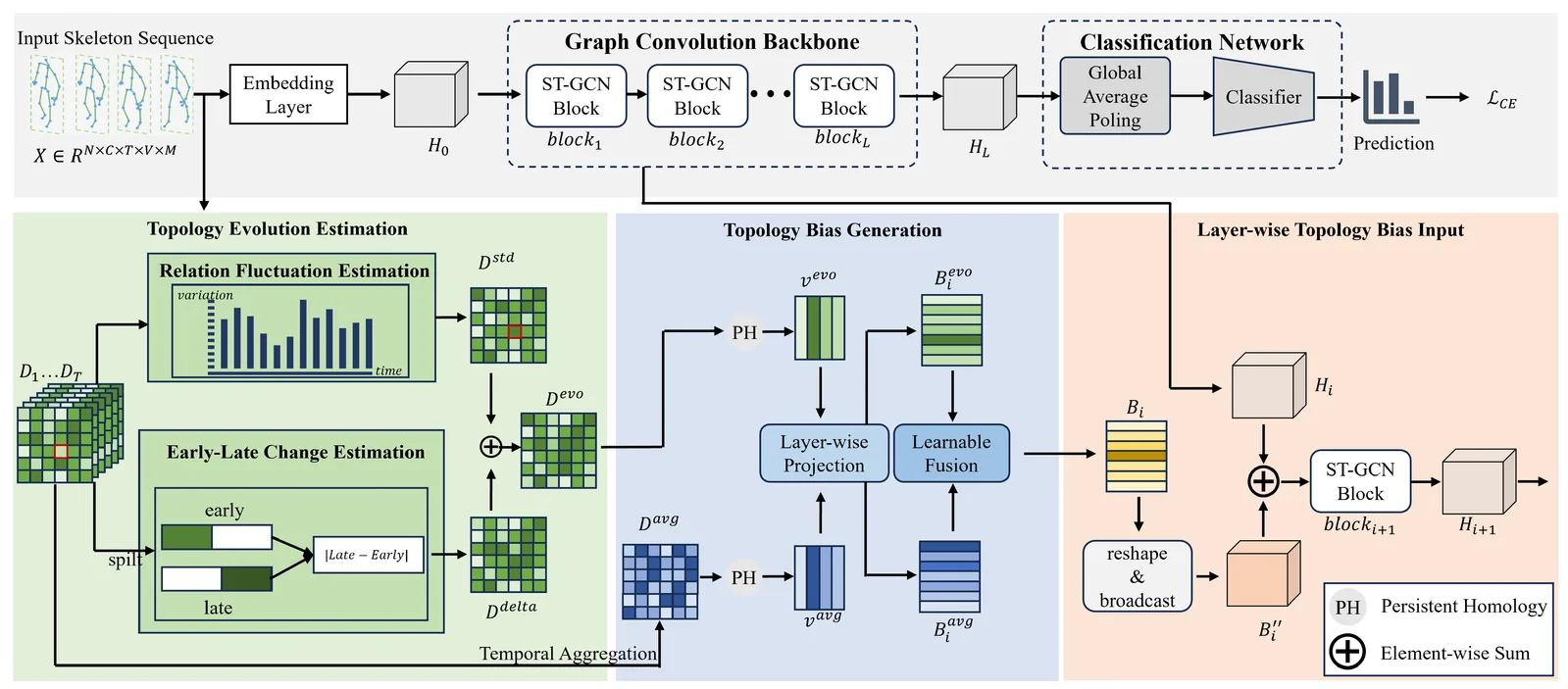
Overall architecture of the proposed JRTE-GCN.

The overall architecture of the proposed method is shown in Figure 1. Given an input skeleton sequence $X\in R^{N\times C\times T\times V\times M}$, where *N*, *C*, *T*, *V*, and *M* denote the batch size, joint coordinate dimension, sequence length, number of joints, and number of persons, respectively, the proposed model consists of four main components: the skeleton action recognition backbone, topology evolution estimation, topology bias generation, and hierarchical topology bias injection.

The input skeleton sequence is fed into both the recognition backbone in the upper branch and the topology evolution estimation module in the lower branch. In the recognition backbone, the original joint coordinates are first passed through an embedding layer to obtain the initial feature representation $H_{0}$, which is then sequentially fed into multiple ST-GCN blocks for spatial-temporal feature extraction. After layer-wise updating through the backbone network, high-level skeleton features $H_{L}$ are obtained and further processed by global average pooling and a classifier to predict the action category.

In the topology evolution estimation module, frame-level joint relation matrices are constructed from the input skeleton sequence. The fluctuation degree of joint relations along the temporal dimension is then measured to obtain the relation fluctuation matrix $D^{\mathrm{std}}$. Meanwhile, the stage variation matrix $D^{\mathrm{delta}}$ is obtained by comparing the joint relation differences between the early and late stages of the action sequence. These two matrices jointly form the topology evolution matrix $D^{\mathrm{evo}}$, which describes the dynamic changes in joint relations during action execution.

The topology bias generation module takes the average topology matrix $D^{\mathrm{avg}}$ and the topology evolution matrix $D^{\mathrm{evo}}$ as inputs. Specifically, $D^{\mathrm{avg}}$ represents the average joint relations over the entire sequence, whereas $D^{\mathrm{evo}}$ describes the temporal evolution of joint relations during the action process. The two matrices are encoded by persistent homology to obtain the average topology descriptor $v^{\mathrm{avg}}$ and the topology evolution descriptor $v^{\mathrm{evo}}$, respectively. Through layer-wise projection, these topology descriptors are mapped into topology biases $B_{i}^{\mathrm{avg}}$ and $B_{i}^{\mathrm{evo}}$, whose channel dimensions are consistent with the backbone features at the corresponding layer. The model then fuses them using learnable residual coefficients to obtain the topology bias $B_{i}$ for the *i*-th layer.

Finally, in the hierarchical topology bias injection module, the topology bias $B_{i}$ of the *i*-th layer is dimensionally aligned and added to the current backbone feature $H_{i}$, serving as the input to the corresponding ST-GCN block. In this way, the recognition backbone can incorporate both the average structural information of the skeleton and the dynamic topology evolution information during layer-wise feature learning.

### 3.2. Topology Evolution Estimation

Given a skeleton sequence, let $x_{t,i}\in R^{C}$ denote the coordinate of the *i*-th joint in the *t*-th frame. The joint relation matrix $D_{t}\in R^{V\times V}$ of the *t*-th frame is defined by the Euclidean distance between each pair of joints:

(1) $$D_{t}\left(i,j\right)={∥x_{t,i}-x_{t,j}∥}_{2},\;i,j=1,\cdots ,V.$$

**Figure 2:**
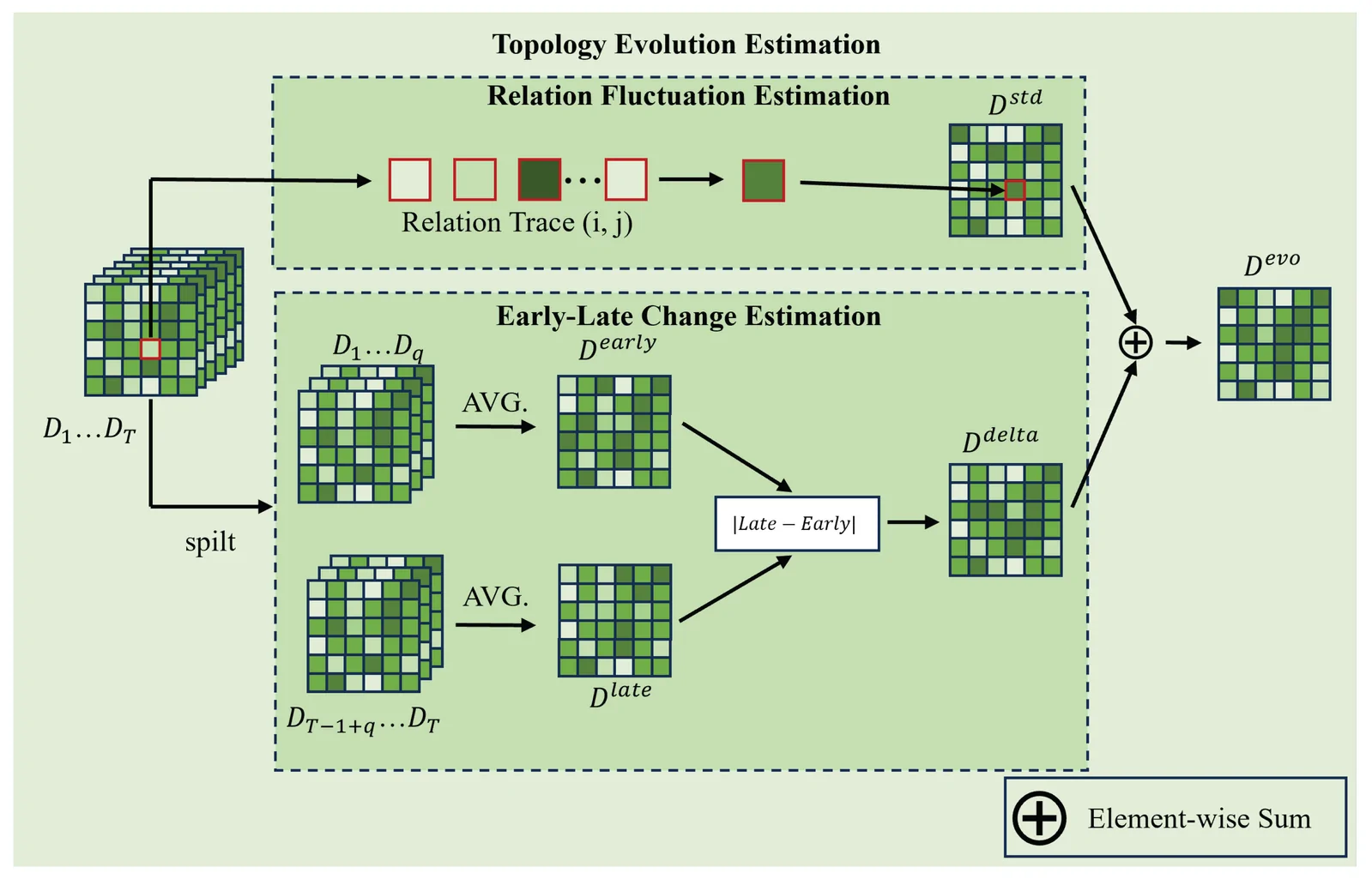
Illustration of the topology evolution estimation module. The relation fluctuation branch computes $D^{\mathrm{std}}$ from temporal relation traces, while the early–late change branch computes $D^{\mathrm{delta}}$ from early and late relation matrices. The two components are normalized and summed to obtain $D^{\mathrm{evo}}$.

As illustrated in Figure 2, the entire action sequence can be represented as a sequence of frame-level joint relation matrices ${\left\{D_{t}\right\}}_{t=1}^{T}$. For any joint pair (i,j), its relation trajectory along the temporal dimension can be written as:

(2) $$R_{i,j}=\left[D_{1}\left(i,j\right),D_{2}\left(i,j\right),\cdots ,D_{T}\left(i,j\right)\right].$$

The relation trajectory $R_{i,j}$ corresponds to the Relation Trace (i,j) in Figure 2, and it is used to describe the distance variation of joint pair (i,j) during action execution.

**Relation fluctuation estimation.** To describe the variation magnitude of joint relations over the entire action sequence, we compute the standard deviation of each joint relation trajectory along the temporal dimension, obtaining the relation fluctuation matrix $D^{\mathrm{std}}$. Let the average relation of joint pair (i,j) over the entire sequence be:

(3) $$\bar{D}\left(i,j\right)=\frac{1}{T}\sum_{t=1}^{T}D_{t}\left(i,j\right).$$

Then, $D^{\mathrm{std}}$ is formulated as:

(4) $$D^{\mathrm{std}}\left(i,j\right)=\sqrt{\frac{1}{T}\sum_{t=1}^{T}{\left(D_{t}\left(i,j\right)-\bar{D}\left(i,j\right)\right)}^{2}}.$$

This matrix describes the fluctuation degree of joint relations during the entire action process. If the distance between a joint pair changes significantly across different frames, the corresponding value of $D^{\mathrm{std}}\left(i,j\right)$ will also be large.

**Stage change estimation.** In addition to the overall fluctuation, we further calculate the joint relation difference between the early and late stages of an action. Let the length of the early and late segments be:

(5) $$q=\max\left(1,\left\lfloor \frac{T}{4}\right\rfloor \right).$$

In practice, the early and late segments are each set to one quarter of the temporally normalized sequence. This choice provides sufficiently long temporal segments to obtain representative early- and late-stage relation statistics while reducing the influence of individual-frame fluctuations. The sensitivity to this segment ratio is further examined in the ablation study.

The joint relation matrices of the first *q* frames and the last *q* frames are averaged to obtain the early-stage relation matrix $D^{\mathrm{early}}$ and the late-stage relation matrix $D^{\mathrm{late}}$, respectively:

(6) $$D^{\mathrm{early}}=\frac{1}{q}\sum_{t=1}^{q}D_{t},$$

(7) $$D^{\mathrm{late}}=\frac{1}{q}\sum_{t=T-q+1}^{T}D_{t}.$$

The stage change matrix $D^{\mathrm{delta}}$ is defined as the element-wise absolute difference between the two matrices:

(8) $$D^{\mathrm{delta}}=\left|D^{\mathrm{late}}-D^{\mathrm{early}}\right|.$$

From a physical perspective, the pairwise relation $D_{t}\left(i,j\right)$ describes the spatial relation between joints *i* and *j* at frame *t*. Variations among the shoulder, elbow, wrist, and hand joints mainly reflect upper-limb motion, whereas variations among the hip, knee, ankle, and foot joints characterize lower-limb motion. Relation changes involving the trunk and multiple limb chains reflect whole-body or postural variation, while relation changes between joints belonging to different body parts provide cues for coordinated motion across body regions. On this basis, $D^{\mathrm{std}}$ measures how strongly these joint relations fluctuate throughout the action sequence, whereas $D^{\mathrm{delta}}$ emphasizes structural changes between the early and late stages of the action. Thus, the proposed representation describes local limb motion, inter-part coordination, and global stage-dependent changes in skeletal configuration.

**Topology evolution matrix construction.** To simultaneously exploit relation fluctuation information and stage change information, we normalize $D^{\mathrm{std}}$ and $D^{\mathrm{delta}}$ separately, and then construct the topology evolution matrix $D^{\mathrm{evo}}$ by element-wise summation:

(9) $$D^{\mathrm{evo}}=N\left(D^{\mathrm{std}}\right)+N\left(D^{\mathrm{delta}}\right).$$

The normalization function N(·) is defined as:

(10) $$N\left(D\right)=\frac{D-\min(D)}{\max(D)-\min(D)+\epsilon }.$$

Here, min(D) and max(D) denote the minimum and maximum values of all elements in matrix *D*, respectively, and ϵ is set to $10^{-6}$ to avoid division by zero.

The normalization is performed independently for each sequence and separately for $D^{\mathrm{std}}$ and $D^{\mathrm{delta}}$ using the minimum and maximum values of the corresponding V×V relation matrix; no batch-level or dataset-level statistics are used.

The resulting $D^{\mathrm{evo}}$ contains both the overall fluctuation information of joint relations and the stage change information between the early and late stages, and is used as the input to the subsequent topology bias generation module.

### 3.3. Topology Bias Generation

**Figure 3:**
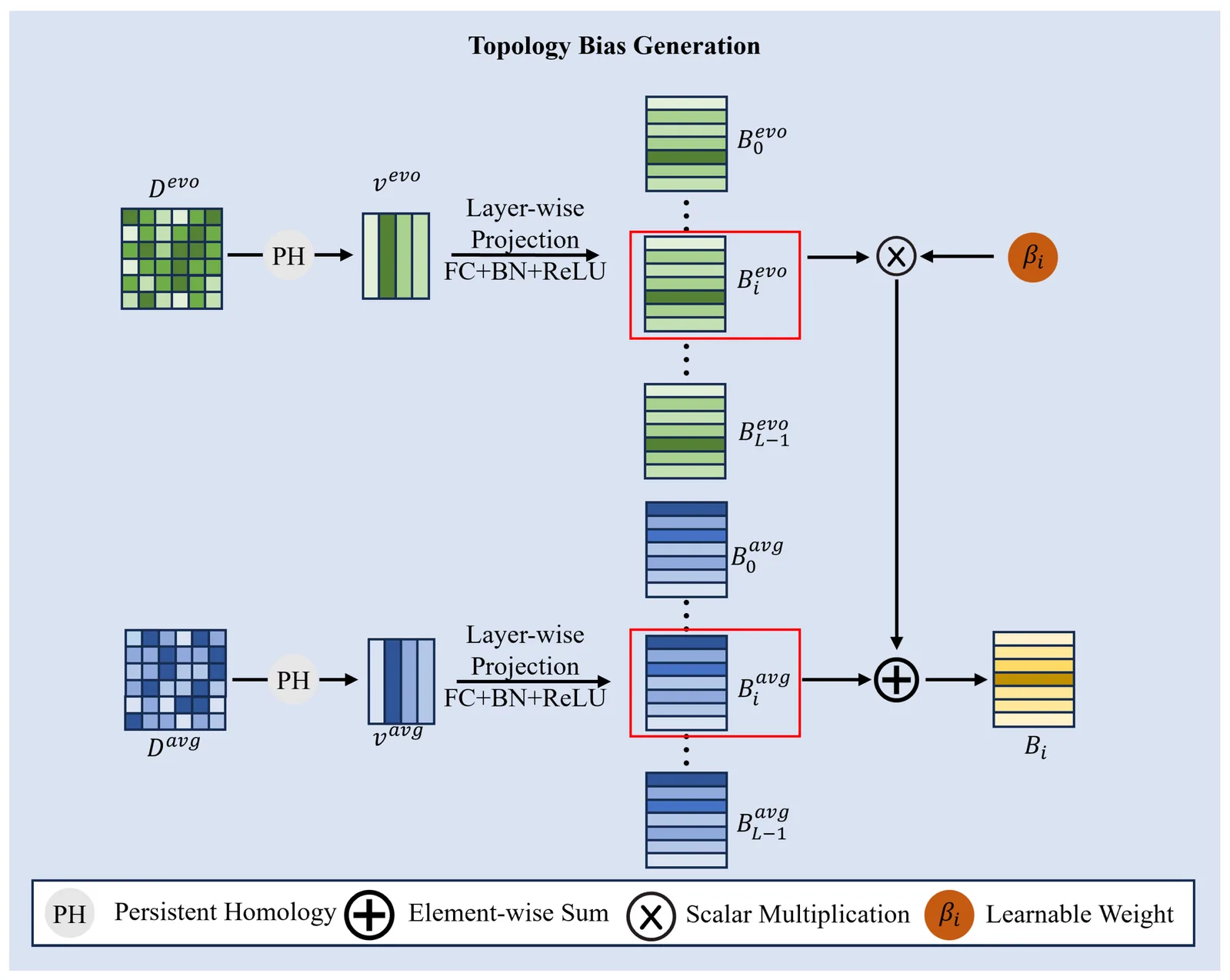
Illustration of the topology bias generation module. The average topology matrix $D^{\mathrm{avg}}$ and the topology evolution matrix $D^{\mathrm{evo}}$ are encoded by persistent homology, projected into layer-wise topology biases, and fused through a learnable residual coefficient to obtain the final topology bias $B_{i}$.

After obtaining the average topology matrix $D^{\mathrm{avg}}$ and the topology evolution matrix $D^{\mathrm{evo}}$, we transform them into topology biases that match the layer-wise feature dimensions of the backbone network. As shown in Figure 3, the overall process consists of three parts: topology encoding, layer-wise projection, and learnable fusion.

**Topology encoding.** The average topology matrix $D^{\mathrm{avg}}$ and the topology evolution matrix $D^{\mathrm{evo}}$ are fed into the persistent homology encoding module to obtain the corresponding topology descriptor vectors $v^{\mathrm{avg}}$ and $v^{\mathrm{evo}}$, respectively:

(11) $$v^{\mathrm{avg}}=\Phi \left(D^{\mathrm{avg}}\right),$$

(12) $$v^{\mathrm{evo}}=\Phi \left(D^{\mathrm{evo}}\right).$$

Here, Φ(·) denotes the persistent-homology-based topology encoding function implemented with torch_topological. For reproducibility, the persistent-homology implementation is pinned to torch_topological v0.1.7. Self-relations are excluded by explicitly setting the diagonal entries of the pairwise relation matrices to zero before persistent-homology encoding. Infinite persistence features are not retained before vectorization, following the default setting keep_infinite_features=False.

The topology matrices are normalized on a per-sequence basis before topological encoding, without using batch-level or dataset-level statistics, consistent with the matrix-wise normalization described in Section 3.2. A Vietoris–Rips filtration is then constructed from the normalized pairwise relation matrix, and zero-dimensional homology ($H_{0}$) is computed to obtain the persistence diagram. The filtration proceeds over the pairwise relation values without imposing an additional finite filtration cutoff.

The persistence diagram is then vectorized by a learnable structural-element layer containing 64 structural elements. For the 64 structural elements, each two-dimensional center is initialized independently from U(0,1), while the corresponding sharpness parameters are initialized to 3. The centers and sharpness parameters are subsequently optimized jointly with the network parameters. Each structural element is parameterized by a learnable center and sharpness and computes an exponential response to the persistence points, with the responses summed over all points to form one descriptor component. The resulting 64 responses constitute the topology descriptor. Accordingly, the average topology and topology evolution descriptors are represented as $v^{\mathrm{avg}},v^{\mathrm{evo}}\in R^{64}$.

**Layer-wise projection.** Since the channel dimensions of backbone features vary across different layers, the topology descriptor vectors need to be mapped into the feature space of each corresponding layer. For the *i*-th layer, $v^{\mathrm{avg}}$ and $v^{\mathrm{evo}}$ are passed through layer-wise projection modules composed of a fully connected layer, batch normalization, and a ReLU activation function, producing the corresponding topology biases $B_{i}^{\mathrm{avg}}$ and $B_{i}^{\mathrm{evo}}$:

(13) $$B_{i}^{\mathrm{avg}}=f_{i}^{\mathrm{avg}}\left(v^{\mathrm{avg}}\right),$$

(14) $$B_{i}^{\mathrm{evo}}=f_{i}^{\mathrm{evo}}\left(v^{\mathrm{evo}}\right).$$

Here, $f_{i}^{\mathrm{avg}}\left(\cdot \right)$ and $f_{i}^{\mathrm{evo}}\left(\cdot \right)$ denote the layer-wise mapping functions for the *i*-th layer. Each projection module follows an FC–BN–ReLU structure. The topology descriptors before projection are 64-dimensional. The projection modules are independently parameterized for different backbone layers and are not shared across layers; moreover, the average-topology and topology-evolution branches use separate projection modules. Specifically, the descriptors are projected from 64 to 128 dimensions for the first five backbone layers and from 64 to 256 dimensions for the remaining five layers, matching the corresponding backbone channel dimensions.

**Learnable fusion.** The average topology bias provides the overall structural information of the entire action sequence, while the topology evolution bias provides the relation change information during the action process. To introduce evolution information while preserving average topology information, we adopt a learnable residual coefficient to fuse the two biases. For the *i*-th layer, the final topology bias $B_{i}$ is defined as:

(15) $$B_{i}=B_{i}^{\mathrm{avg}}+\beta_{i}B_{i}^{\mathrm{evo}}.$$

Here, $\beta_{i}\in R$ denotes a learnable scalar for the *i*-th backbone layer. Each layer has its own independent coefficient, which controls the contribution of the topology evolution bias in that layer. All $\beta_{i}$ are initialized to 0.005 and jointly optimized with the other network parameters. No explicit constraint, clipping operation, or additional regularization is applied to $\beta_{i}$ during training. The fused $B_{i}$ is then used as the topology bias of the *i*-th layer.

### 3.4. Layer-Wise Topology Bias Injection

After obtaining the topology bias $B_{i}$ for the *i*-th layer, JRTE-GCN injects it into the corresponding ST-GCN block by adding it to the current backbone feature. Let the feature at the *i*-th layer be $H_{i}\in R^{NM\times C_{i}\times T_{i}\times V}$, where $C_{i}$, $T_{i}$, and *V* denote the channel dimension, temporal length, and number of joints, respectively.

Since $B_{i}$ contains channel-wise topology bias information, it is first reshaped into $B_{i}^{\prime }\in R^{NM\times C_{i}\times 1\times 1}$ and then broadcast along the temporal and joint dimensions:

(16) $$B_{i}^{\prime \prime }\in R^{NM\times C_{i}\times T_{i}\times V}.$$

The expanded topology bias is added to the backbone feature to obtain the topology-enhanced feature:

(17) $${\tilde{H}}_{i}=H_{i}+B_{i}^{\prime \prime }.$$

The enhanced feature is then fed into the next ST-GCN block:

(18) $$H_{i+1}={\mathrm{Block}}_{i+1}\left({\tilde{H}}_{i}\right).$$

Through this layer-wise topology bias injection strategy, average topology information and topology evolution information are continuously fused with backbone features at different depths. This enables the model to exploit both stable skeletal structure and dynamic joint-relation changes during spatial-temporal feature extraction.

## 4. Experiments

### 4.1. Datasets

We train and evaluate the proposed method on three benchmark datasets for skeleton-based action recognition, namely NTU RGB+D 60, NTU RGB+D 120, and Northwestern-UCLA [27,28,29]. NTU RGB+D 60 is one of the most widely used benchmark datasets for skeleton-based action recognition. It contains 60 action classes performed by 40 subjects from three camera views, with a total of 56,880 action samples. Each skeleton sample is represented by 25 anatomically defined joints. Following the common settings in previous studies, this dataset is evaluated under two protocols: Cross-Subject (X-Sub) and Cross-View (X-View), which are used to assess the generalization ability of the model across different subjects and camera views, respectively.

NTU RGB+D 120 is an extended version of NTU RGB+D 60 and follows the same 25-joint skeleton definition. It contains 120 action classes, 106 subjects, and 114,480 action samples. This dataset is usually evaluated under two protocols: Cross-Subject (X-Sub) and Cross-Setup (X-Set). Northwestern-UCLA is a commonly used multi-view skeleton-based action recognition dataset. In our experiments, each Northwestern-UCLA skeleton is represented by 20 joints. It contains 10 action classes performed by 10 subjects from three camera views. Following the standard cross-view evaluation protocol, the first two views are used for training, while the third view is used for testing.

For clarity, the joint indices follow the standard anatomical definitions of the corresponding skeleton formats. For NTU RGB+D 60/120, joints 1–25 correspond to SpineBase, SpineMid, Neck, Head, ShoulderLeft, ElbowLeft, WristLeft, HandLeft, ShoulderRight, ElbowRight, WristRight, HandRight, HipLeft, KneeLeft, AnkleLeft, FootLeft, HipRight, KneeRight, AnkleRight, FootRight, SpineShoulder, HandTipLeft, ThumbLeft, HandTipRight, and ThumbRight, respectively. For Northwestern-UCLA, joints 1–20 correspond to HipCenter, Spine, ShoulderCenter, Head, ShoulderLeft, ElbowLeft, WristLeft, HandLeft, ShoulderRight, ElbowRight, WristRight, HandRight, HipLeft, KneeLeft, AnkleLeft, FootLeft, HipRight, KneeRight, AnkleRight, and FootRight, respectively. Accordingly, the indices *i* and *j* in $D_{t}\left(i,j\right)$ directly refer to the corresponding dataset-specific joint indices defined above.

### 4.2. Implementation Details

The model is optimized using stochastic gradient descent (SGD) with Nesterov momentum, where the momentum parameter is set to 0.9. Cross-entropy loss is used in all experiments. All models are trained for 140 epochs with a 5-epoch warm-up. The initial learning rate is set to 0.05 and is decayed by a factor of 0.1 at epochs 110 and 120, respectively. For the NTU RGB+D 60 and NTU RGB+D 120 datasets, the weight decay is set to 0.0004, the batch size is set to 64, and the temporal length of each sample is uniformly adjusted to 64 frames. For the Northwestern-UCLA dataset, the weight decay is set to 0.0002, and the batch size is set to 16. The persistent-homology descriptors are computed online during forward propagation rather than precomputed offline.

For NTU RGB+D 60/120, valid skeleton sequences are temporally cropped and resized to 64 frames. Random 3D rotation is applied during training as data augmentation, while dataset-level coordinate normalization is disabled in the feeder used in our experiments. No additional explicit view-alignment module is applied. The topology branch constructs pairwise joint distances from the temporally processed joint-coordinate sequences. For Northwestern-UCLA, the skeleton sequence contains 20 joints and is sampled to 52 frames; the coordinates are centered and normalized to [−1,1], with random view rotation and scaling applied during training and uniform temporal sampling used during testing.

The experiments are conducted on Ubuntu 20.04 with Python 3.8, PyTorch 1.10.0, and CUDA 11.3. All experiments are mainly performed on an NVIDIA RTX 4090D GPU, with an 18-vCPU AMD EPYC 9754 128-Core Processor as the CPU configuration.

To quantify the additional computational overhead introduced by topology evolution modeling, the baseline and JRTE-GCN are compared under the same Joint single-stream configuration and hardware environment. The results are summarized in Table 1. For computational-cost evaluation, parameter counts include all trainable parameters. FLOPs are calculated for a single forward pass using a batch size of 1 and an NTU RGB+D input sequence of 64 frames. Inference latency is measured with a batch size of 1 using the same 64-frame input length. Before timing, 50 forward passes are performed as warm-up, followed by 200 timed forward passes. The function torch.cuda.synchronize() is called immediately before and after each timed forward pass to ensure completion of all CUDA operations. The reported inference latency is the arithmetic mean over the 200 timed runs. Peak GPU-memory usage and training time per epoch are measured using the training batch size of 64 under the same hardware environment.

As shown in Table 1, JRTE-GCN increases the parameter count from 1.30 M to approximately 1.50 M and the computational cost from 1.63 GFLOPs to approximately 1.64 GFLOPs. The peak GPU memory usage increases from approximately 5.5 GB to 6.0 GB, while the training time increases from approximately 6.3 to 6.8 min per epoch. The average inference latency increases from approximately 2.4 to 2.6 ms per sample. The additional overhead mainly arises from online persistent-homology encoding and the layer-wise projection of topology descriptors. Overall, the additional computational cost remains moderate relative to the baseline.

### 4.3. Comparison with Existing Methods

To evaluate the performance of JRTE-GCN, we compare it with representative skeleton-based action recognition methods on NTU RGB+D 60, NTU RGB+D 120, and Northwestern-UCLA under their standard evaluation protocols.

JRTE-GCN adopts four input modalities, namely Joint (J), Bone (B), Joint Motion (JM), and Bone Motion (BM).

The final prediction score is obtained by a weighted sum of the class scores produced by the four independently trained streams,

(19) $$s_{\mathrm{fuse}}=w_{J}s_{J}+w_{B}s_{B}+w_{\mathrm{JM}}s_{\mathrm{JM}}+w_{\mathrm{BM}}s_{\mathrm{BM}},$$

where $s_{J}$, $s_{B}$, $s_{\mathrm{JM}}$, and $s_{\mathrm{BM}}$ denote the class-score vectors of the Joint, Bone, Joint Motion, and Bone Motion streams, respectively.

For NTU RGB+D 60/120, the fusion weights for J, B, JM, and BM are 0.60, 0.65, 0.35, and 0.20, respectively; for Northwestern-UCLA, the corresponding weights are 0.70, 0.50, 0.60, and 0.20. Since the compared methods employ different input configurations, the input setting corresponding to each reported result is explicitly specified in Table 2. Unreported results are denoted by “—”.

As shown in Table 2, JRTE-GCN achieves accuracies of 93.3%, 97.2%, 90.5%, 91.7%, and 97.0% under the five evaluation settings, respectively. These results are obtained using the four-stream fusion of Joint, Bone, Joint Motion, and Bone Motion inputs. JRTE-GCN achieves the best results on NTU RGB+D 60 X-Sub and both protocols of NTU RGB+D 120, while remaining competitive on NTU RGB+D 60 X-View and Northwestern-UCLA. Together with the explicitly reported input settings in Table 2, these results provide a clearer basis for comparison with existing multi-stream and ensemble-based methods.

### 4.4. Ablation Study

To verify the effectiveness of different components in JRTE-GCN, ablation experiments are primarily conducted on NTU RGB+D 60 under the X-Sub protocol using the Joint single-stream input. Unless otherwise specified, the same data preprocessing, backbone, optimizer, learning-rate schedule, and training epochs are retained, and only the module or parameter setting under evaluation is changed. Additional results on NTU RGB+D 120 X-Sub and repeated-run statistics are reported to evaluate cross-dataset consistency and run-to-run consistency, respectively.

#### 4.4.1. Construction of Topology Evolution Information

To further evaluate different dynamic-relation modeling strategies under controlled conditions, we compare the baseline and several relation-evolution representations using the same backbone, Joint single-stream input, and training configuration. The final JRTE-GCN models topology evolution using $D^{\mathrm{std}}$ and $D^{\mathrm{delta}}$. As an alternative comparison variant, we additionally introduce an adjacent-frame relation variation matrix $D^{\mathrm{diff}}$, defined as

(20) $$D^{\mathrm{diff}}=\frac{1}{T-1}\sum_{t=1}^{T-1}\left|D_{t+1}-D_{t}\right|,$$

where $D_{t}$ denotes the pairwise joint-relation matrix at frame *t*. The $D^{\mathrm{diff}}$ variant is used only for comparison and is not included in the final JRTE-GCN configuration.

For all five settings, the backbone and Joint single-stream input are unchanged, and identical preprocessing, optimizer, learning-rate schedule, and 140-epoch training schedule are used; only the temporal joint-relation representation is varied.

As shown in Table 3, the baseline achieves accuracies of 90.8% and 86.9% on NTU RGB+D 60 and NTU RGB+D 120 X-Sub, respectively. The alternative $D^{\mathrm{diff}}$ representation achieves 90.9% and 87.0%, while $D^{\mathrm{std}}$ achieves 91.1% and 87.3%, and $D^{\mathrm{delta}}$ achieves 91.0% and 87.1%. Combining $D^{\mathrm{std}}$ and $D^{\mathrm{delta}}$ yields the highest accuracies of 91.2% and 87.4% on the two datasets, respectively.

The results show consistent trends across the two datasets and indicate that different temporal relation representations capture complementary dynamic information. $D^{\mathrm{diff}}$ mainly reflects local frame-to-frame relation changes, whereas $D^{\mathrm{std}}$ characterizes relation fluctuations over the entire sequence and $D^{\mathrm{delta}}$ captures structural differences between the early and late stages of an action. The proposed combination of $D^{\mathrm{std}}$ and $D^{\mathrm{delta}}$ achieves the best performance on both datasets.

#### 4.4.2. Stability Analysis

To further evaluate the stability of the performance gain, the baseline and JRTE-GCN are each evaluated over three runs with different random seeds on NTU RGB+D 60 under the X-Sub protocol using the Joint single-stream input. The results are reported in Table 4.

As shown in Table 4, JRTE-GCN achieves a mean accuracy of 91.20%, compared with 90.80% for the baseline. Both settings exhibit small standard deviations, while JRTE-GCN maintains an average improvement of 0.40 percentage points across the repeated runs. Under the tested NTU RGB+D 60 X-Sub setting with the Joint single-stream input, JRTE-GCN consistently outperforms the baseline across the three tested random seeds. This repeated-seed observation is limited to the above experimental setting and is not generalized to other datasets, evaluation protocols, or the four-stream fusion configuration without additional repeated-seed experiments.

#### 4.4.3. Sensitivity to the Segment Ratio

To examine the influence of the segment length used in stage change estimation, we vary the segment ratio q/T while keeping the remaining settings unchanged. The experiments are conducted on NTU RGB+D 60 under the X-Sub protocol using the Joint single-stream input.

As shown in Table 5, the recognition accuracy varies within a relatively narrow range from 90.98% to 91.20% across the tested segment ratios. The best performance is obtained at q/T=1/4. Accordingly, q/T=1/4 is adopted as the default segment ratio in JRTE-GCN.

#### 4.4.4. Effect of the Residual Fusion Coefficient

The residual fusion coefficient $\beta_{i}$ controls the initial input strength of the topology evolution branch. To examine its influence, the initial value of $\beta_{i}$ is set to 0, 0.001, 0.002, 0.005, 0.007, and 0.01, respectively, while all other settings remain unchanged. The results are shown in Table 6.

As shown in Table 6, the model achieves the highest accuracy of 91.20% when $\beta_{i}$ is initialized to 0.005. A proper non-zero initialization allows topology evolution information to participate in feature updating from the early training stage. However, larger initial values do not further improve performance, and the accuracy decreases to 91.02% and 90.87% when $\beta_{i}$ is initialized to 0.007 and 0.01, respectively. Therefore, the default initial value of $\beta_{i}$ is set to 0.005.

#### 4.4.5. Analysis of Topology Evolution Information Input Positions

To analyze the effect of input positions, topology evolution information is injected into different network layers, including single-layer positions and continuous layer regions. The results are shown in Table 7.

As shown in Table 7, single-layer input brings limited gains, with accuracies around 90.91–90.92%. Continuous-layer input performs better, indicating that topology evolution information is more effective when it participates in feature transformation across adjacent layers. The best result of 91.20% is achieved when topology evolution information is injected into all layers, suggesting that it provides complementary guidance at different feature depths. Therefore, JRTE-GCN adopts all-layer input in the final setting.

### 4.5. Visualization Analysis

**Figure 4:**
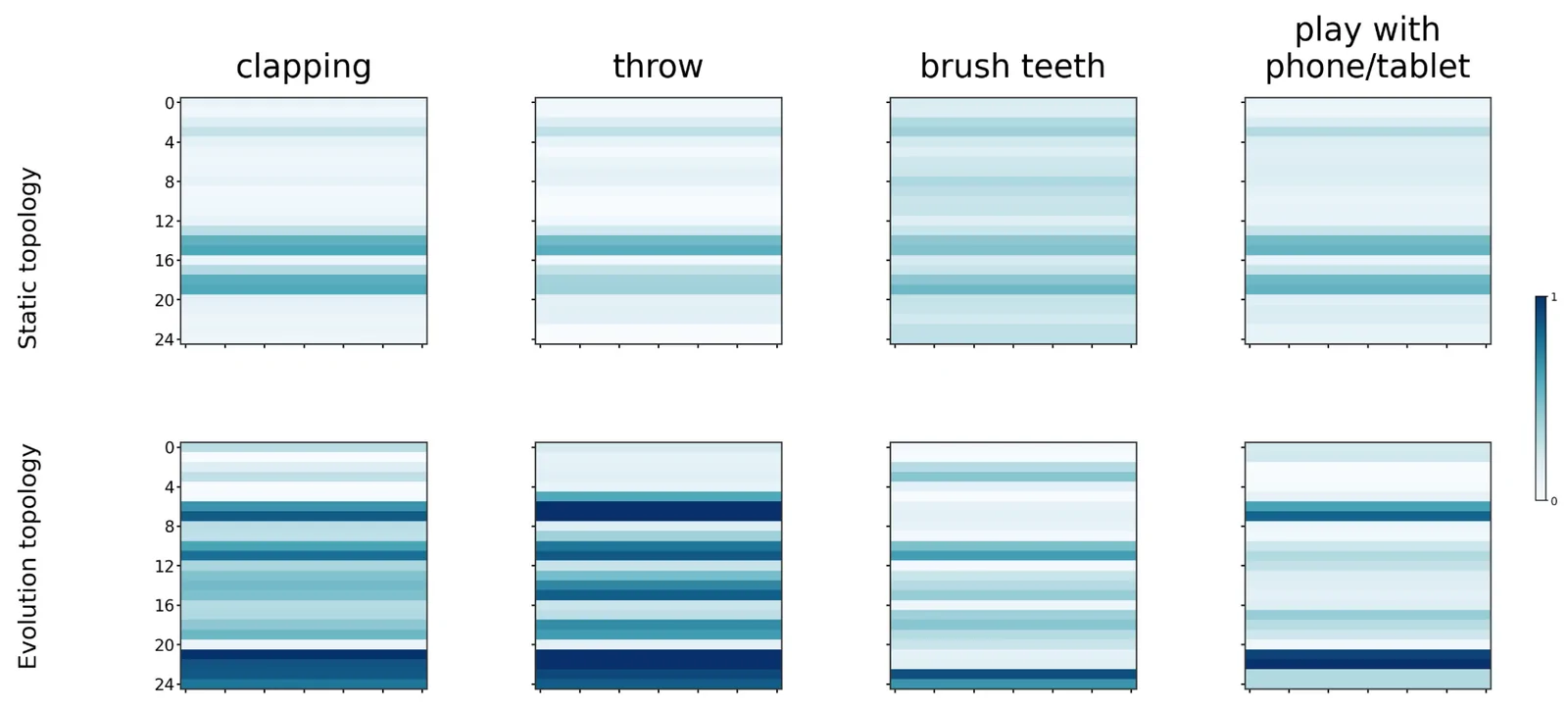
Visualization of static topology and topology evolution responses for representative action classes.

To further verify the effectiveness of the proposed topology modeling strategy, we visualize topology responses and class-wise accuracy changes. As shown in Figure 4, static topology presents relatively consistent responses across different action categories, indicating its role in providing stable skeletal structural priors. In contrast, topology evolution produces stronger and more category-dependent responses. For actions involving frequent hand or upper-limb movements, such as *clapping*, *throw*, and *play with phone/tablet*, the responses become more prominent around upper-limb and distal-joint regions, while *brush teeth* shows a more local response distribution. These results indicate that topology evolution can adaptively emphasize action-related dynamic joint relations.

From the perspective of anatomical joint groups, different skeleton nodes and their pairwise relations are expected to characterize complementary types of action-related behavior. Upper-limb joints mainly describe arm and hand motion, while lower-limb joints reflect locomotion-related changes involving the hips, knees, ankles, and feet. Coordinated changes involving the trunk and multiple limb chains characterize whole-body posture variation. Moreover, pairwise relations among joints provide cues for inter-joint coordination across different body parts. In the temporal dimension, the early–late relation difference $D^{\mathrm{delta}}$ further emphasizes structural changes between different stages of an action. Therefore, the proposed topology evolution representation captures both local limb motion and global structural changes during action execution.

**Figure 5:**
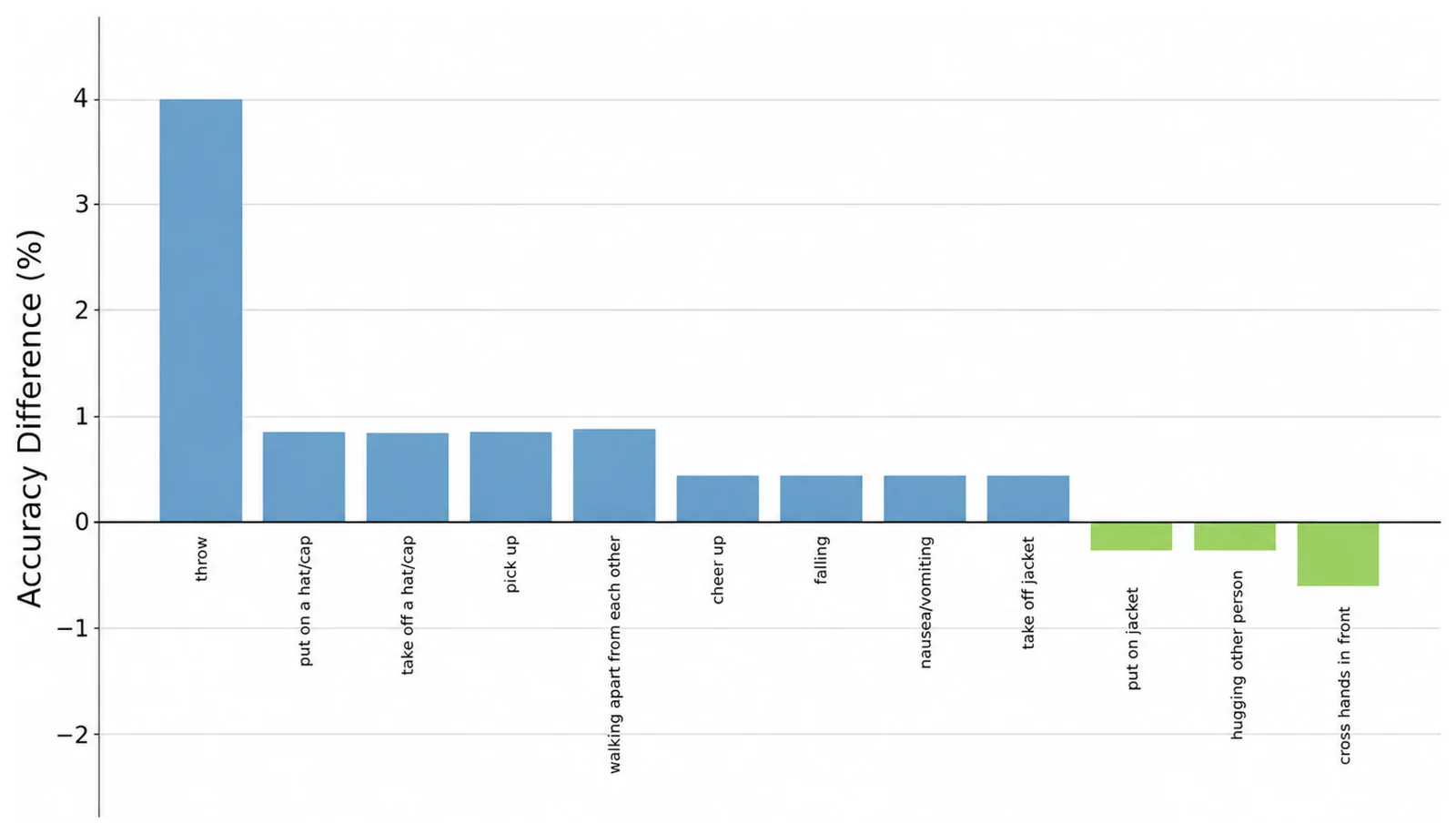
Class-wise accuracy gains of JRTE-GCN over the baseline on the top 12 action classes with the strongest joint-relation evolution.

To evaluate topology evolution at the category level, the top 12 categories with the strongest relation evolution are selected to compare class-wise accuracy changes, as shown in Figure 5. The *throw* category obtains the largest performance gain. This action typically involves pronounced upper-limb swinging, trunk-driven motion, and directional changes, which lead to evident temporal variations in inter-joint relations and therefore benefit more from topology evolution modeling. In addition, categories such as *put on a hat/cap*, *take off a hat/cap*, *pick up*, and *walking apart from each other* also obtain improvements, covering local upper-body motion, whole-body posture change, lower-limb motion, and human-interaction behavior.

In contrast, categories such as *cross hands in front*, *hugging other person*, and *put on jacket* show different degrees of performance degradation. These actions often involve local body-part overlap, self-occlusion, or similar poses across categories. In such cases, strengthening dynamically varying joint relations may also amplify local interference or non-discriminative relation changes, thereby reducing the stability of category discrimination. This observation reveals a limitation of the current topology evolution representation for actions with severe local overlap or high inter-class pose similarity.

Overall, topology evolution is most effective for actions with pronounced temporal structural changes, coordinated limb motion, and evident stage-wise relation variations. In contrast, it is less effective for actions with subtle inter-class differences, severe local overlap, self-occlusion, or highly similar poses. The topology response distributions and class-wise accuracy changes therefore provide complementary evidence for both the effectiveness and the limitations of the proposed topology evolution modeling strategy.

## 5. Conclusions

This paper proposes JRTE-GCN, a graph convolutional network framework that integrates topology evolution information into skeleton-based action recognition. Different from methods that rely only on fixed skeletal connections or static joint relation modeling, the proposed framework models both the temporal fluctuations of joint relations and the structural differences between the early and late stages of an action, encodes the resulting topology evolution information through persistent homology, and transforms the extracted descriptors into layer-wise topology biases for graph convolutional feature updating. Therefore, the primary contribution of this work lies in the overall framework for topology evolution modeling and bias injection, rather than in any individual temporal statistic alone.

The proposed method is evaluated on standard benchmark datasets for skeleton-based action recognition and compared with the baseline and existing methods. The experimental results show that JRTE-GCN achieves incremental but generally consistent improvements over the baseline and competitive performance compared with existing methods. Further ablation studies verify the effectiveness of topology evolution modeling and the topology bias injection module, demonstrating that dynamic topology information provides a useful complement to the average topology representation. The magnitude of the performance gains varies across datasets, evaluation protocols, and action categories, which is consistent with the incremental improvements observed in the experiments.

The ability to characterize dynamic joint-relation changes also gives the proposed framework potential value in practical human-motion understanding scenarios.

More specifically, the extracted temporal structural information describes changes in inter-joint relations, coordinated body-part motion, and stage-wise skeletal configurations. Such information is potentially relevant to rehabilitation and healthcare assessment for characterizing changes in movement patterns and coordination, to human–robot interaction for representing evolving human actions, and to industrial safety and surveillance for describing motion patterns associated with human activities. It may also provide structural motion cues for sports-motion analysis and assisted-living scenarios, where temporal coordination and action-stage transitions are relevant. These application scenarios are discussed as potential directions only; no application-specific experiments are conducted in this study, and no performance claims are made for these practical settings.

In future work, we will further explore more efficient ways to compute topological features to reduce the additional computational cost introduced by topology modeling. Meanwhile, we will also consider extending the proposed topology evolution modeling strategy to more input modalities and more complex action recognition scenarios, aiming to further improve the generalization ability and practical application value of the model.

## Figures and Tables

**Table 1:** Computational-cost comparison between the baseline and JRTE-GCN under the Joint single-stream setting.

Method	Params (M)	GFLOPs	Memory (GB)	Train (min/Epoch)	Inference (ms/Sample)
Baseline	1.30	1.63	∼5.5	∼6.3	∼2.4
JRTE-GCN	1.50	1.64	∼6.0	∼6.8	∼2.6

**Table 2:** Recognition accuracy (%) comparison on three benchmark datasets.

Methods	Publication	Input	NTU RGB+D 60		NTU RGB+D 120		NW-UCLA
			X-Sub	X-View	X-Sub	X-Set	Acc.
ST-GCN [11]	AAAI 2018	J	81.5	88.3	70.7	73.2	—
2s-AGCN [12]	CVPR 2019	2S	88.5	95.1	82.5	84.2	—
MS-G3D [16]	CVPR 2020	2S	91.5	96.2	86.9	88.4	—
MST-GCN [30]	AAAI 2021	4S	91.5	96.6	87.5	88.8	—
CTR-GCN [18]	ICCV 2021	4S	92.4	96.8	88.9	90.6	96.5
EfficientGCN-B4 [20]	TPAMI 2022	MIB	91.7	95.7	88.3	89.1	—
InfoGCN [19]	CVPR 2022	6S	93.0	97.1	89.8	91.2	97.0
GAP [31]	ICCV 2023	4S	92.9	97.0	89.9	91.1	**97.2**
DS-GCN [32]	AAAI 2024	4S	93.1	**97.5**	89.2	91.1	—
BlockGCN [26]	CVPR 2024	4S	93.1	97.0	90.3	91.5	96.9
DG-STGCN(S0.8) [33]	CVPR 2025	4S	92.8	97.3	89.0	90.7	—
FD-GCN [34]	Pattern Recognition 2026	4S	92.8	96.7	89.4	90.7	97.0
JRTE-GCN	Ours	4S	**93.3**	97.2	**90.5**	**91.7**	97.0

J denotes joint input; 2S denotes the two-stream fusion of joint and bone inputs (J + B); 4S denotes the four-stream fusion of joint, bone, joint-motion, and bone-motion inputs (J + B + JM + BM); 6S denotes the six-stream ensemble setting reported for InfoGCN; and MIB denotes the early-fused multiple-input-branch setting of EfficientGCN-B4 using joint, velocity, and bone inputs. Bold indicates the best result. — indicates unreported results.

**Table 3:** Dynamic-relation modeling comparison on NTU RGB+D 60/120 X-Sub using Joint single-stream input.

Setting	Accuracy (%)	
	NTU60 X-Sub	NTU120 X-Sub
Baseline	90.8	86.9
$D^{\mathrm{diff}}$	90.9	87.0
$D^{\mathrm{std}}$	91.1	87.3
$D^{\mathrm{delta}}$	91.0	87.1
$D^{\mathrm{std}}+D^{\mathrm{delta}}$	**91.2**	**87.4**

**Table 4:** Stability analysis on NTU RGB+D 60 X-Sub using the Joint single-stream input.

Setting	Run 1	Run 2	Run 3	Mean ± Std.
Baseline	90.73	90.82	90.85	90.80±0.06
JRTE-GCN	91.14	91.21	91.25	91.20±0.06

**Table 5:** Sensitivity analysis of the segment ratio q/T on NTU RGB+D 60 under the X-Sub protocol using the Joint single-stream input.

Segment Ratio q/T	Accuracy (%)
1/8	91.02
1/6	91.11
1/4	**91.20**
1/3	91.13
1/2	90.98

**Table 6:** Ablation results of different initial values of the residual fusion coefficient $\beta_{i}$.

Initial Value of $\beta_{i}$	0	0.001	0.002	0.005	0.007	0.01
Accuracy (%)	90.90	90.93	90.97	**91.20**	91.02	90.87

**Table 7:** Ablation study of topology evolution information input positions on NTU RGB+D 60 under the X-Sub protocol using the Joint single-stream input.

Setting	Injected Layers	Accuracy (%)
Single early layer	i=1	90.91
Single middle layer	i=5	90.92
Single late layer	i=10	90.92
Early layers	i=1,…,4	90.94
Middle layers	i=4,…,7	90.96
Late layers	i=7,…,10	90.95
All layers	i=1,…,10	91.20

## Data Availability

The datasets used in this study are publicly available benchmark datasets. NTU RGB+D 60 and NTU RGB+D 120 are available from the original dataset providers, and Northwestern-UCLA is also publicly available from the corresponding dataset source. The data used to support the findings of this study can be accessed through these public dataset repositories.
